# Association between HLA molecules and stage III/IV grade C periodontitis in a Moroccan population: A case-control study

**DOI:** 10.34172/japid.026.3839

**Published:** 2026-03-16

**Authors:** Khadija Amine, Wafa El Kholti, Siham Bennani, Jamila Kissa

**Affiliations:** ^1^Periodontics Department, Faculty of Dentistry, University Hassan II of Casablanca, Casablanca, Morocco; ^2^Pasteur Institute of Casablanca, Morocco

**Keywords:** HLA antigens, Genetics, Periodontitis, Risk

## Abstract

**Background.:**

Literature data support the important role played by HLA molecules in predisposing to periodontitis. The present study evaluated possible associations between HLA class I and II phenotypes and stage III/IV grade C periodontitis in a Moroccan population.

**Methods.:**

The sample comprised 26 patients aged 15‒30 years, diagnosed with stage III/IV grade C periodontitis (case group) and 202 patients ethnically similar to the case group (control group). The patients underwent phenotyping for HLA class I molecules using the BIOTEST microlymphocytic plate technique and genotyping for HLA DR molecules. Bivariate analysis was performed using the chi-squared test at a significance level of *P*<0.05.

**Results.:**

The results showed a variable distribution of A alleles, without any specificity, predominantly in periodontitis cases compared with the controls. B21 allele was found with a lower phenotypic frequency (10.34%) in periodontitis cases compared with controls (31.18%) (χ^2^=5.40 and *P*=0.02). The phenotypic frequency of the DR6 allele was significantly lower in periodontitis cases (11.52%) compared with the controls (33.16%) (χ^2^=5.07 and *P*=0.024).

**Conclusion.:**

The present case‒control study reported a negative association between B21 and DR6 alleles and stage III/IV grade C Moroccan periodontitis cases compared with the controls with verified healthy periodontal status. This may help explain the particular profile of stage III/IV grade C periodontitis in Moroccan patients.

## Introduction

 The human leucocyte antigen (HLA) system is a multigene, multiallelic, codominant system that plays a role in the initiation of specific immune responses. It consists of a set of genes located on a segment of the short arm of chromosome 6. The loci are essentially grouped into two classes:

- Class I: It includes HLA-A, B, and C genes and is related to cellular immunity.
- Class II: It has three main sub-regions: DR, DP, and DQ, and is related to humoral immunity.

The molecules of the HLA system play a crucial role in distinguishing self from non-self. Polymorphism of these molecules is involved in the specific immune response through antigen presentation and T cell selection. As a result, susceptibility and resistance to certain diseases depend essentially on the different HLA antigens (HLA class I or II) expressed on the cell surface.

 Thus, certain HLA genotypes have been strongly associated with certain diseases, in particular autoimmune diseases.^1^ Some HLA haplotypes have been associated with susceptibility and others with resistance to periodontal diseases.^1‒3^

 In Caucasian populations, the HLA-A9 and HLA-B15 alleles are associated with a higher risk of periodontal destruction.^3^ Some studies have shown a statistically significant association between HLA-B15 antigens and aggressive periodontitis (AgP) (stage III/IV grade C).^4^ A relationship has also been observed between HLA class II molecules (HLA DR) and susceptibility to AgP (stage III/IV grade C), in particular HLA-DR4.^5^ A study by Takashiba et al.^6^ suggested the involvement of the HLA DRB1*1501-DGB1*0602 region in susceptibility to AgP (stage III/IV grade C) in a Japanese population.^6^

 More recently, a meta-analysis by Stein et al.^7^ showed that HLA-A9 and HLA-B15 were associated with AgP (stage III/IV grade C), and HLA-A2 and HLA-B5 were protective factors against periodontal disease in a Caucasian population.

 All these data support the important role played by HLA molecules in predisposing to stage III/IV grade C periodontitis.

 However, results from other studies on the association between HLA specificities and susceptibility or resistance to a disease have shown that this association depends on the distribution of these specificities in the studied population.

 The prevalence of periodontitis has been well assessed in Morocco.^8‒10^ The authors reported that the prevalence of staged periodontitis in the young population was among the highest reported in worldwide national studies. 12.3% (or almost 360.894 subjects) had periodontitis. 6.1% of Moroccan young subjects have stage III/IV periodontitis.

 In this context, our work focused on identifying possible associations between HLA class I and II phenotypes and stage III/IV grade C periodontitis in a Moroccan population (previously termed AgP).

## Methods

###  Participants

####  Case Group

 The case group consisted of 26 consenting, healthy, non-smoking patients aged 15‒30 years, diagnosed with stage III/IV grade C periodontitis.^11,12^ Patients were recruited from the Ibn Rochd University Hospital Center, Casablanca, over a period of 8 months. None of the participants had undergone any periodontal treatment or antibiotic therapy within 3 months before the study.

####  Control Group

 A second group of 202 consenting, periodontally healthy, non-smoking patients aged 15‒30 years, ethnically similar to the case group and selected from the same population, served as the control group.

 Both groups had the same sex and demographic characteristics.

###  Clinical and Radiographic Examination

 Clinical examination used the Williams periodontal probe to measure probing depth and clinical attachment loss at 6 sites per tooth (distobuccal, midbuccal, mesiobuccal, distopalatal, midpalatal, and mesiopalatal). Full-mouth x-rays, including 14 retro-alveolar radiographs, were taken for all the participants. To avoid inter-examiner error, one calibrated periodontist (KA) performed clinical and radiographic examinations of all the participants.

###  Biological Processing and Microscopic Analysis

 The patients underwent phenotyping for HLA class I molecules using the BIOTEST microlymphocytic plate technique. Genotyping using a DYNAL kit was used to determine DRB alleles by hybridizing amplified DNA with probes specific to HLA-DRB loci attached to a nylon membrane.

###  Typing of HLA A and B Molecules Using the Microlymphocytoxicity Technique

 The technique is a two-step reaction. The specific antibody (serum from an anti-HLA-immunized subject) is incubated with the antigen (lymphocytes to be typed), and rabbit complement is then added to the reaction.

 If the lymphocytes carry the HLA antigen recognized by the serum, the cell lysis reaction takes place and is visualized by staining.

 Test serumsare generally obtained from multiparous women. They are selected and prepared by dilution to have the following characteristics: they are monospecific or bispecific and yield reproducible, contrasting results.

 BIOTEST plates sensitized with the anti-sera panel were used. The suspension was then adjusted to a cell suspension of 2.500 lymphocytes per µL and incubated for 30 minutes at 22°C with monoclonal antibodies labelled with fluorescein (FITC) or phycoerythrin (PE). 1 μL of serum was injected into each alveolus; a 30-minute cell antigen incubation was carried out at 22°C; then 6 mL of rabbit complement was added to the wells and incubated for 90 minutes at 22°C. The plates were then emptied of their anti-serum.

 The plates were then emptied of their contents, and 1 µL of trypan blue solution diluted 1:2 in hypertonic HANKS medium was added to the wells.

 Readings were taken within minutes at the end of incubation using an inverted microscope equipped with a phase-contrast device.

 The percentage of dead cells reflected the reaction’s intensity.

 For each cell, the results were recorded on a protocol sheet on which the specificity of the sera was clearly listed for each cell:

 0% dead cells = 0

 0‒20% reaction = 2

 20‒50% = 4

 50‒80% = 6

 80‒100% = 8

###  Typing of HLA and DR Molecules Using Polymerase Chain Reaction (PCR)

 The typing of alleles at the HLA DR locus uses molecular biological techniques, the major advantage of which is the direct study of polymorphic genes.

 After genomic DNA extraction, a polymerase chain reaction (PCR) was performed using a biotinylated primer specific for the HLA-DRB gene*.

 The amplification product was then detected by hybridization to a nylon membrane bearing oligoprobes specific for the HLA DRB1*, DRB2*, DRB4*, and DRB5* loci. The streptavidin biotin-HRP complex was revealed using a chromogenic substrate (hydrogen peroxide/tetramethylbenzidine).

 The results were derived from an interpretation table listing the possible combinations of the DRB gene’s alleles.

###  Statistical Analysis 

 Qualitative variables were expressed as percentages. Bivariate analysis was performed using the chi-squared test with a significance level of P < 0.05.

###  Ethical Review

 The study protocol was approved by the Pedagogic Committee and the Research Conduct Committee of the Faculty of Dentistry, Hassan II University of Casablanca.

## Results

 The phenotypic frequencies of the HLA-A, HLA-B, and HLA-DR loci found in periodontitis cases and controls are shown in Tables 1, 2, and 3.

 The results of this analysis showed a variable distribution of A alleles, without any specificity observed in periodontitis cases compared with controls. However, 20.68% of cases could not be characterized in terms of HLA-A because the panel of allelic specificities available to us did not correspond to any specificity in our population.

 For HLA-B molecules, the distribution of alleles in our periodontitis patients did not show any increase in the phenotypic frequency of antigenic specificities compared with controls.

 Nevertheless, analysis of the results showed an absence of the B18, B22, B37, B70, and B78 alleles without this difference being significant between periodontitis cases and the controls (RR < 2). B21 allele was found with a lower phenotypic frequency (10.34%) in periodontitis cases compared with controls (31.18%), (χ^2^ = 5.40 and *P* = 0.02).

 Regarding HLA-DR molecules, typing was performed by molecular biology, and showed the presence of all alleles in both periodontitis cases and control groups. No predominance of a special allele was noted in both groups. On the other hand, the phenotypic frequency of the DR6 allele was significantly lower in periodontitis cases (11.52%) compared with controls (33.16%) (^2^ = 5.07 and *P* = 0.024).

 The study of the HLA/periodontitis association in our population did not identify a specific allele associated with the disease for either HLA-A or HLA-DR molecules.

**Table 1:** Results of phenotypic frequencies of HLA A molecules in periodontitis cases and healthy controls

**Allele**	**Cases**	**Controls**	**χ2 **	***P***	**OR**
	**Phenotypic frequency** **(%)**	**Phenotypic frequency** **(%)**			
A1	10.34	21.78	2.047	0.152	0.414
A2	20.68	32.66	1.697	0.192	0.538
A3	13.78	18.8	0.429	0.512	0.690
A9	27.58	33.16	0.36	0.548	0.768
A10	10.34	16.32	0.692	0.405	0.591
A11	10.34	4.44	1.706	0.181	2.482
A19	48.26	47.52	0.534	0.44	1.030
A28	34.48	25.24	1.113	0.199	1.559

*HLA: human leucocyte antigen; OR: odds ratio

**Table 2:** Results of phenotypic frequencies of HLA B molecules in periodontitis cases and healthy controls

**Allele**	**Cases**	**Controls**	**χ** ^ 2 ^	***P***	**OR**
	**Phenotypic frequency** **(%)**	**Phenotypic frequency** **(%)**			
B5	27.58	18.3	1.39	0.239	1.700
B7	13.78	19.3	0.158	0.691	0.668
B8	3.44	12.86	2.18	0.140	0.241
B12	24.12	32.66	0.855	0.355	0.655
B13	10.34	7.42	0.301	0.583	1.439
B14	17.24	11.88	0.664	0.415	1.557
B16	10.34	16.32	0.692	0.405	0.591
B17	10.34	11.38	0.275	0.686	0.898
B21	10.34	31.18	5.4	0.020^*^	0.255
B27	6.88	3.96	0.528	0.468	1.792
B35	6.88	11.88	0.631	0.427	0.548
B40	6.88	3.46	0.797	0.372	2.061
B41	3.44	1.98	0.258	0.611	1.764

*HLA: human leucocyte antigen; OR: odds ratio

**Table 3:** Results of phenotypic frequencies of HLA DR molecules in periodontitis cases and controls

**Allele**	**Cases**	**Controls**	**χ** ^ 2 ^	***P***	**OR**
	**Phenotypic frequency** **(%)**	**Phenotypic frequency** **(%)**			
DR1	7.69	18.3	2.36	0.125	0.372
DR2	38.46	29.2	0.914	0.339	1.515
DR3	7.69	23.26	4.07	0.04	0.283
DR4	38.46	21.78	3.65	0.056	2.244
DR5	11.52	24.24	2.81	0.093	0.407
DR6	11.52	33.16	6.25	0.012*	0.262
DR7	38.46	36.62	0.018	0.889	1.082
DR8	3.84	3.46	0.00	0.996	1.114
DR9	3.84	1.98	0.258	0.611	1.977
DR10	15.38	7.92	1.11	0.293	2.113

*HLA: human leucocyte antigen; OR: odds ratio

## Discussion

 The results of the present case‒report study showed that B21 and DR6 alleles appear to be significantly lower in Moroccan stage III/IV grade C periodontitis cases compared with controls (*P* = 0.02 and *P* = 0.024, respectively).

 Among the genetic factors incriminated in the pathogenesis of periodontitis, major histocompatibility complex (MHC) molecules play a crucial role. In this context, the HLA genes encoding the molecules that present antigenic peptides to specific T lymphocytes have been studied.

 No previous data are available regarding the association between HLA molecules and periodontitis in Moroccan patients.

 The very high allelic variability of HLA genes could influence the specificity and the type of immune response to various antigens, leading to individual differences in susceptibility to infectious diseases. Amine et al.^13^ noted in their case‒control study of Moroccan AgP patients a significantly reduced expression of ICAM-1 (CD54) molecules, which could affect cell adhesion, mobility, and intercellular interactions. Another case‒control study performed by the same authors showed that patients with AgP produced statistically more IL-10 and less INF- than healthy patients.^14^ The production of these inflammatory mediators could be affected by different genetic traits.^15^

 Susceptibility to periodontal inflammation is influenced by epigenetic regulation. The epigenetic methylation in the pathway of the Toll-like receptors (TLR) could affect the induction or prevention of localized AgP.^16^

 The contribution of HLA genes to periodontal infections has been reported in various studies and affects both HLA class I and class II molecules. Takashiba et al.^6^, in a Japanese population, found a significant increase in the DRB1*1501 and DRB1*0602 alleles in periodontitis patients. More recently, Shimomura-Kuroki et al.^17^ reported a positive association between the HLA DQB1 allele and localized AgP in another Japanese population. More recently, Mateuella et al.^18^ analyzed HLA-G polymorphisms in AgP, chronic periodontitis (ChP), and healthy Brazilian patients. The authors showed that ChP patients had significantly increased homozygosity for the 14 bp deletion allele. Sippert et al.^19^, in their case‒control study conducted on ChP Brazilian patients, demonstrated that HLA A2 and B40 could be associated with ChP. However, HLA B15 and DRB1∗11 could contribute to the resistance to ChP in the Brazilian population. Stein et al.^3^, in a study of a German periodontitis population, suggested that HLA-A9 and HLA-B15 alleles could be considered risk factors for periodontitis. These data confirm the results of a meta-analysis conducted by the same authors who showed that HLA A9 and B15 were associated with AgP. At the same time, HLA A2 and B5 were likely protective factors against AgP.^7^ Mauramo et al.^1^, in their cross-sectional study of a Swiss population, reported that HLA B15, B51, and DRB1*12 were associated with fewer periodontal manifestations. Mausavi et al.^20^, in a case‒control study on an Iranian AgP population, showed a statistically significant association with HLA DRB1*04:01, HLA DQA1*03:01, HLA DQB1*03:02, HLA DRB1*16:01, HLA DQA1*01:03, and HLA DQB1*05:01 haplotypes compared to a control group. More recently, in the same population, Mausavi et al.20 reported that HLA-Cl II polymorphisms were associated with resistance to and susceptibility to AgP.

 It may be hypothesized that heterogeneity in published data reflects racial differences in HLA allelic frequencies.

 Our study showed a variable distribution of HLA class I (A and B) and class II (DR) molecules in the case group, without any particular allele being preferentially associated with periodontitis. However, the B21 and DR6 alleles appear to be weakly expressed in our patients compared with controls, with a statistically significant difference (*P* = 0.02 and *P* = 0.024, respectively). It may suggest a protective association between B21 and DR6 alleles and stage III/IV grade C periodontitis.

 It was difficult to conclude whether B21 and DR6 alleles are involved in periodontitis resistance. The recognized protection of an allele requires a periodontally healthy control population that is presumed to be resistant to the disease. These data were not verified in our control population.

 A large sample and a periodontally healthy control population would be necessary to better study the associations between stage III/IV grade C periodontitis and HLA molecules.

## Conclusion

 The present case‒control study reported a negative association between B21 and DR6 alleles and stage III/IV grade C periodontitis cases in Morocco compared with controls with verified healthy periodontal status. To the best of our knowledge, this association has not been reported in other populations. This may help explain the particular profile of stage III/IV grade C periodontitis in Moroccan patients.

## Competing Interests

 The authors declare that they have no competing interests regarding authorship and/or publications of this paper.

## Data Availability

 All data generated and analyzed during this study are included in this manuscript.

## Ethical Approval

 The protocol of the study was approved by the Pedagogic Committee and the Research Conduct Committee of the Faculty of Dentistry, Hassan II University of Casablanca.

 Date of approval: December 24^th^, 2024.
